# Cortical Frontoparietal Network Dysfunction in *CHMP2B*-Frontotemporal Dementia

**DOI:** 10.3389/fnagi.2021.714220

**Published:** 2021-09-13

**Authors:** Christian Sandøe Musaeus, Jette Stokholm Pedersen, Troels Wesenberg Kjær, Peter Johannsen, Gunhild Waldemar, Maria Joy Normann Haverberg, Theis Bacher, Jørgen Erik Nielsen, Peter Roos, The FReJA Consortium, S Gydesen

**Affiliations:** Danish Dementia Research Centre (DDRC), Rigshospitalet and Department of Clinical Medicine, University of Copenhagen, Copenhagen, Denmark; Psychiatric Centre Ballerup, Copenhagen University Hospital, Ballerup, Denmark; Department of Neurology, Addenbrooke’s Hospital, Cambridge, United Kingdom; Department of Neurodegenerative Disease and UK Dementia Research Institute at UCL, UCL Queen Square Institute of Neurology, London, United Kingdom; Institute of Prion Diseases and MRC Prion Unit at UCL, Courtauld Building, London, United Kingdom; Department of Psychology, University of Copenhagen, Copenhagen, Denmark; Department of Pathology, University Hospital, Lund, Sweden; Parkvaenget Nursing Home, Holstebro, Denmark; Department of Pathology, Aalborg University Hospital, Aalborg, Denmark; ^1^Danish Dementia Research Centre (DDRC), Rigshospitalet and Department of Clinical Medicine, University of Copenhagen, Copenhagen, Denmark; ^2^Department of Neurology, Zealand University Hospital, Roskilde, Denmark; ^3^Department of Clinical Medicine, University of Copenhagen, Copenhagen, Denmark

**Keywords:** EEG, microstates, spectral power, FTD, Frontotemporal dementia, CHMP2B, microstates analysis

## Abstract

A rare cause of inherited frontotemporal dementia (FTD) is a mutation in the *CHMP2B* gene on chromosome 3 leading to the autosomal dominantly inherited FTD (*CHMP2B*-FTD). Since *CHMP2B*-FTD is clinically well-characterized, and patients show a distinct pattern of executive dysfunction, the condition offers possible insight in the early electroencephalographic (EEG) changes in the cortical networks. Specifically, EEG microstate analysis parses the EEG signals into topographies believed to represent discrete network activations. We investigated the EEG dynamics in patients with symptomatic *CHMP2B*-FTD (*n* = 5) as well as pre-symptomatic mutation carriers (*n* = 5) compared to non-carrier family members (*n* = 6). The data was parsed into four archetypal microstates and global power was calculated. A trend was found for lower occurrence in microstate D in *CHMP2B*-FTD (*p-*value = 0.177, *F-*value = 2.036). Patients with recent symptom onset (<1 year) showed an increased duration of microstate D, whereas patients who had been symptomatic for longer periods (>2 years) showed decreased duration. Patients with *CHMP2B*-FTD present with executive dysfunction, and microstate D has previously been shown to be associated with the fronto-parietal network. The biphasic pattern may represent the pathophysiological changes in brain dynamics during neurodegeneration, which may apply to other neurodegenerative diseases.

## Introduction

Frontotemporal dementia (FTD) is a common cause of early-onset dementia (Ratnavalli et al., 2002), and disease-causing mutations have been identified in a number of genes (Rohrer et al., 2009; Paulson and Igo, 2011). One of these genes is *CHMP2B* on chromosome 3 causing an autosomal dominantly inherited FTD previously named FTD-3, now *CHMP2B*-FTD, which has only been described in one large family globally. It is caused by a single base change, c.532-1G > C in the *CHMP2B* gene (Skibinski et al., 2005). The phenotype of FTD-3 patients is behavioral-variant-FTD, most often characterized by initial impairment in executive functions (working memory, attentional control, inhibition and mental flexibility) as the first and most pronounced symptom (Stokholm et al., 2013). Looking at the structural brain changes using MRI, a longitudinal study showed increased atrophy rates in the inferior temporal cortex, superior frontal cortex and the insular cortex when comparing mutation carriers to non-carriers (Eskildsen et al., 2009). In addition, when investigating metabolic changes as measured by 18F-fluro-deoxy-glucose positron emission tomography (FDG-PET), involvement of the parietal areas was shown in pre-symptomatic mutation carriers, while changes in frontal areas appeared once the subjects became symptomatic (Johannsen et al., 2016). Despite a low sample size due to the rarity of the disease, the patients were all from the same family, and no studies have so far investigated the functional brain dynamics using quantitative electroencephalography (qEEG) in patients with *CHMP2B*-FTD.

In a transgenic mouse model of CHMP2B, a study has found short and long-range synaptic degeneration (Ghazi-Noori et al., 2012) and studies have also applied qEEG as a way to investigate functional brain dynamics (Stothart et al., 2016; Tait, 2019). Here, one study found that the changes were most pronounced the frontal areas (Tait, 2019) and suggested a compensatory mechanism of local synchrony to compensate for the breakdown of the cortical networks.

One type of qEEG marker that has been applied is spectral power or the squared amplitude of EEG rhythmic signal. Here, in patients with behavioral-variant FTD, studies have found conflicting results with some reporting an increased slowing (Stigsby et al., 1981; Besthorn et al., 1996; Yener et al., 1996; Passant et al., 2005), while another study found an absence of increased slow-wave activity, but a decrease of fast-wave activity (Lindau et al., 2003). In support of the latter, a recent study found that the peak frequency was not as affected in patients with FTD as seen in patients with AD (Goossens et al., 2017).

A method to study cortical brain networks using EEG is microstate analysis. This technique involves segmenting the EEG signal into a number of states (Lehmann et al., 1987) defined by spatial topographies. These functional states have been shown to be reliable over multiple recordings (Khanna et al., 2014). Furthermore, microstates have been linked to activity of resting state networks as measured with resting state fMRI (Van De Ville et al., 2010; Yuan et al., 2012). In FTD, a study investigated the changes in microstates in behavioral variant FTD and found changes in microstate C (Nishida et al., 2013), which has been linked to the insular-cingulate network (Britz et al., 2010). This finding may explain the changes in personality such as disinhibition and apathy. Since executive function is the main cognitive domain in *CHMP2B*-FTD, other cortical networks may be affected.

In the current exploratory study, we wanted to investigate changes in global brain dynamics using spectral power and microstates in a cohort of *CHMP2B* mutation carriers. This is unique since no study has investigated quantitative EEG markers in a genetically uniform cohort of FTD patients.

## Materials and Methods

### Study Population

The Danish *CHMP2B*-FTD family has been subject to extensive studies within the Frontotemporal dementia in Jutland Association (FReJA) collaboration (Gydesen et al., 2002). Clinical characteristics have been recorded in 45 cases of disease, providing information about disease course and neuropsychological changes. EEGs were obtained from a total of 16 participants: five symptomatic patients with *CHMP2B*-FTD, five pre-symptomatic *CHMP2B* mutation carriers, and six non-carrier family members (controls). As a measure of global cognitive function, Addenbrooke’s cognitive examination (ACE) (Mathuranath et al., 2000) was performed on all patients and three of the pre-symptomatic carriers. As it is impossible to define an exact time of onset, disease duration was based on the time where a close relative first noticed symptoms. All participants provided written informed consent. The study was approved by the Ethics Committee of the Capital Region of Denmark (H-1-2012-041) and all methods were performed in accordance with the regulations.

### EEG Recordings

EEG data were acquired at 200 Hz and recorded to a TrackIt Mk3^TM^ (Lifelines Neurodiagnostic Systems, Illinois, United States) using WaveGuard caps with Ag/AgCl electrodes (ANT Neuro). This equipment was chosen since it was possible to bring along for recording at patients’ homes. The following channels were used: Fp1, Fp2, F7, F3, Fz, F4, F8, C3, C4, T3, T5, T4, T6, P3, Pz, P4, O1, and O2. The impedance before recording was below 10 kOhm or if it was not possible to reach below 10 kOhm, the channel was noted for the subsequent pre-processing. The EEGs were recorded, alternating between 30 s periods each of eyes closed and eyes open. All participants were able to perform this task.

### Pre-processing EEG

For the subsequent pre-processing and analysis, we used MATLAB (Mathworks, v2017b) and EEGLAB toolbox v13.6.5b (Delorme and Makeig, 2004). First, the data were imported, and the eyes-closed segments of EEGs were selected. To computationally locate the electrodes on the scalp, we used the DIPFIT toolbox (Oostenveld et al., 2011). The data were bandpass-filtered from 1–70 Hz using the *pop_firws* function in MATLAB with a filter order of two, and the Kaiser window parameter beta was estimated using a maximum passband ripple of 0.001. Afterward, the data was band-stop filtered from 45–55 Hz using the same settings. Next, the data were divided into 3-s epochs as has previously been applied, and the EEGs were visually inspected to remove excessive noise or artifacts. If one or more channels contained excessive noise, drift or a bad connection, they were interpolated using spherical interpolation. In no participants were more than three electrodes interpolated. Afterward, the EEGs were re-referenced to average reference (using the *fullRankAveRef* function), and independent component analysis was performed using the extended infomax algorithm (Lee et al., 1999). This was done for each file, and components which mostly contained eye movement or ECG artifacts, were removed. A maximum of two components were removed for each subject. The investigator was blinded to CHMP2B mutation and clinical status.

### Spectral Power Calculations

To calculate spectral power, we calculated power across epochs using the in-built function *spectopo* from the EEGLAB toolbox. The window length was equal to the sampling rate of 200 Hz. The relative power was calculated in each of the following frequency bands: delta (1–3.99 Hz), theta (4–7.99 Hz), alpha (8–12.99 Hz), and beta (13–29.99 Hz) by dividing the power of each frequency bands with the total power. Afterward, we calculated global relative power by averaging across all channels for each frequency band for each subject. Absolute power was not included in the current analysis due to the large variability between subjects (Musaeus et al., 2018).

### Microstates Analysis

The microstate analysis was performed using the Microstate EEGlab Toolbox (Poulsen et al., 2018). First, the EEGs were lowpass-filtered at 40 Hz. For each subject we began by extracting the first 2,500 electric field montages at global field power (GFP) peaks with a minimum peak distance of 10 ms. GFP peaks that exceeded two times the standard deviation of the GFPs of all maps were excluded. To identify topographic clusters within these data, we submitted all *n* × 2,500 electric potential topographies to a modified K-means clustering algorithm (Pascual-Marqui et al., 1995) using 300 repetitions. Polarity of the EEG topography was ignored (Lehmann, 1971; Wackermann et al., 1993; Pascual-Marqui et al., 1995). We chose to predefine the number of microstates as four to remain consistent with the majority of prior studies of EEG microstates (Khanna et al., 2014), and because four has been confirmed to generate reproducible maps (Khanna et al., 2015). A set of four global maps was generated (see Supplementary Figure 2) and back fitted to the whole EEG. To reduce noise, we rejected microstate segments shorter than 30 ms. This step was performed since we assumed that shorter segments were due to noise. After back-fitting the global maps, we calculated global explained variance (GEV) as defined in the Microstate EEGlab Toolbox (Poulsen et al., 2018), duration, occurrence, and coverage for EEG files. Here, the duration was defined as the average time for each map to be present before transitioning to another map. Occurrence was defined as the average number of times a microstate occurred each second. Coverage was defined as the total percent of the EEG for which a microstate was accounted for. GEV was defined as the variance of EEG activity explained by all four microstates.

### Statistics

All statistics were performed in MATLAB (vR2017b). To compare gender, we performed Fisher’s exact test due to the low sample size. The tests were performed between controls and pre-symptomatic carriers, between controls and *CHMP2B*-FTD, and between pre-symptomatic carriers and *CHMP2B*-FTD. For age and number of epochs, we performed a Kruskal–Wallis test, and for comparing ACE between pre-symptomatic carriers and patients, we used Wilcoxon rank sum test.

When comparing relative power and microstate features between subjects, we first log-transformed the data due to the non-normal distribution. Although not significantly different between the groups, gender and age have been shown to affect microstates features (Tomescu et al., 2018) and were therefore used as covariates in the ANCOVA (Gruner, 2020) analyses. In addition, we performed ANCOVA analysis between the patients and controls using the same covariates as mentioned above. Lastly, to understand the effect size, we calculated Cohen’s d for the occurrence of microstate D between patients and controls with the data not being log-transformed.

Due to low sample size, we did not perform any statistical analysis when comparing patients with short duration of symptoms (<1 year), and patients who had been symptomatic for a longer period (>2 years).

## Results

### Demographics

Demographics are listed in Table 1. No significant difference in age was found between controls, pre-symptomatic carriers and patients (*p-*value = 0.110) although pre-symptomatic carriers were generally younger than clinically affected patients. There were no significant differences in gender between the three groups. The average disease duration for *CHMP2B*-FTD was 3.4 years (standard deviation: 2.73). We did not find a significant difference in the number of 3-s epochs (*p*-value = 0.807).

**TABLE 1:** Characteristics of study participants.

	**Controls**	**Pre-symptomatic carriers**	**CHMP2B-FTD**	***p*-value**
Age, mean (SD)	55.17 (10.34)	42.40 (16.86)	63.80 (9.01)	0.110
Gender, females/males	3/3	3/2	2/3	1.000
ACE, mean (SD)	N/A	89.00 (8.64)	68.80 (7.70)	0.107
Number of epochs, mean (SD)	54.67 (10.31)	52.80 (18.85)	50.00 (10.22)	0.807

*FTD3, Frontotemporal dementia type 3; ACE, Addenbrooke’s cognitive examination. *P*-values for age, gender and number of epochs show the differences when comparing patients with CHMP2B-FTD, pre-symptomatic carriers, and controls. When investigating the differences in ACE, only two pre-symptomatic carriers had an ACE performed while no controls had an ACE performed. Therefore, we used Wilcoxon rank sum test.*

### Spectral Power

We did not find any significant differences in the electrode-to-electrode comparisons. Furthermore, we did not find any significant differences in global relative power, but there was a trend toward an increase in relative alpha power for patients and especially for pre-symptomatic mutation carriers (*p*-value = 0.099, *F*-value = 2.881) as compared to controls (see Supplementary Figure 3).

### Microstates

There was no significant difference in the total GEV between controls, pre-symptomatic mutation carriers and patients (*p*-value = 0.271, *F*-value = 1.446), with an average GEV across groups of 49.67%. No significant differences were found for the microstate features between the three groups, see Table 2. However, a trend was found for lower occurrence of microstate D in pre-symptomatic carriers and patients compared to healthy controls (*p*-value = 0.177, *F*-value = 2.036). Between symptomatic and controls we also found a trend for the occurrence of microstate D (*p*-value = 0.064) and a large effect size as measured with Cohen’s d (*d* = 0.940).

**TABLE 2:** Microstate features, including duration, occurrence, and coverage for microstates A-D, and the *p*-value and *F*-values comparing FTD-3, pre-symptomatic carriers, and HC.

	**Duration**					**Occurence**					**Coverage**				
	**Controls**	**Pre-symptomatic**	**CHMP2B-FTD**	***p*-value**	***F*-value**	**Controls**	**Pre-symptomatic**	**CHMP2B-FTD**	***p*-value**	***F*-value**	**Controls**	**Pre-symptomatic**	**CHMP2B-FTD**	***p*-value**	***F*-value**
Microstate A	77.75 (4.56)	86.11 (11.05)	89.85 (19.44)	0.381	1.06	2.80 (0.46)	2.52 (0.50)	2.79 (0.91)	0.781	0.25	21.76 (3.54)	21.55 (4.01)	25.75 (12.15)	0.819	0.20
Microstate B	77.48 (4.97)	94.83 (16.83)	79.67 (8.65)	0.276	1.45	2.73 (0.40)	2.86 (0.23)	2.72 (0.52)	0.986	0.01	21.25 (4.02)	27.01 (5.03)	21.40 (3.44)	0.383	1.05
Microstate C	82.91 (8.27)	81.95 (16.44)	84.90 (9.61)	0.732	0.32	3.14 (0.51)	2.53 (0.72)	2.79 (0.60)	0.396	1.01	26.13 (5.85)	21.24 (9.35)	23.85 (6.40)	0.563	0.61
Microstate D	91.92 (10.86)	100.48 (27.15)	98.90 (38.15)	0.953	0.05	3.36 (0.16)	3.05 (0.40)	2.88 (0.73)	0.177	2.04	30.86 (3.67)	30.21 (6.51)	28.99 (13.54)	0.737	0.31

**P*-values show the differences when comparing patients with CHMP2B-FTD, pre-symptomatic carriers, and controls.*

**FIGURE 1:**
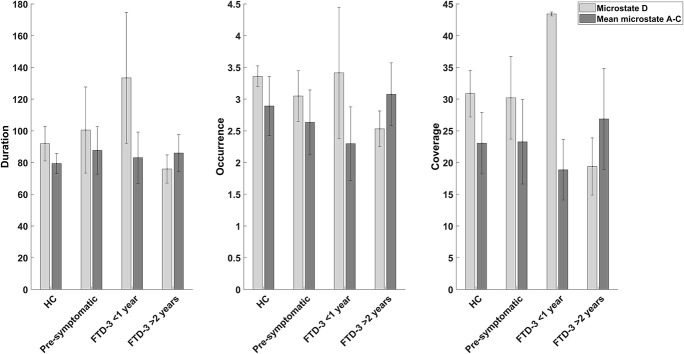
Bar graphs showing the mean and standard deviation of duration, occurrence, and coverage for microstate D and the mean of the other microstates (A, B, and C) for the controls (*n* = 6), pre-symptomatic carriers (*n* = 5), patients with CHMP2B-FTD with recent (<1 year) onset of symptoms (*n* = 2), and patients who had been symptomatic for a longer (>2 years) period (*n* = 3).

When investigating the difference between microstate features between the two patients with short duration of symptoms (<1 year), and the three patients who had been symptomatic for a longer period (>2 years), we found an increase in features for microstate D and decreased coverage for the rest of the microstates (see Figure 1 and Supplementary Table 4).

## Discussion

In the current exploratory study, we investigated the changes in global brain dynamics using spectral power and microstates in a cohort of patients with *CHMP2B*-FTD. A trend was found between the three groups for occurrence of microstate D (*p*-value = 0.177, *F*-value = 2.036) with a large effect size (*d* = 0.940) between the patients and controls. When dividing the patients based on disease duration, we found a biphasic pattern for microstate D with increased microstate features in patients with recent onset of symptoms (<1 year), while patients who had been symptomatic for longer (>2 years) showed decreased network activity in microstate D. This finding suggests an increased activity of the underlying network in the early stages of the disease. Looking at relative power, we found a trend in global relative power in the alpha band, which was most pronounced in pre-symptomatic carriers (*p-*value = 0.099, *F-*value = 2.881).

One study has investigated changes in microstates in patients with behavioral variant FTD and found the duration of microstate C significantly lower compared to controls (Nishida et al., 2013). Microstate C has previously been associated with the insular-cingulate network (Britz et al., 2010), which has been shown to be affected in behavioral variant FTD (Zhou et al., 2010). In our current study we found that microstate D showed a trend toward a decrease in *CHMP2B*-FTD as compared to controls. A possible explanation for these conflicting findings could be that patients with behavioral variant FTD usually present with changes in personality and behavior, while patients with *CHMP2B*-FTD initially present with executive dysfunction while behavioral symptoms appear at a later stage of the disease (Stokholm et al., 2013). To understand the connection between microstates and the spatial changes in the networks, studies have investigated the connection between microstate D and both the blood oxygen level-dependent signal and resting state networks measured with fMRI (Britz et al., 2010; Musso et al., 2010; Yuan et al., 2012) as well as using source localization (Custo et al., 2017). For all the aforementioned methods, microstate D has been associated with the fronto-parietal network, which has been shown to be related to executive function (Reineberg and Banich, 2016). Our finding that patients with *CHMP2B*-FTD show a distinct pattern of disruption of the microstate D is in accordance with a frontoparietal dysfunction presenting as the clinical feature of reduced executive function.

Since our participants are all from the same family, and the *CHMP2B*-FTD is due to a specific genetic mutation, we would have expected that all patients showed a decrease in microstate D features. However, diverging results have also been found in studies of EEG microstates for microstate A in patients with Alzheimer’s disease. Here, some studies comparing Alzheimer’s disease and controls found a shorter average duration of microstates in patients with Alzheimer’s disease (Dierks et al., 1997; Strik et al., 1997; Stevens and Kircher, 1998), but this may be due to using temporal windowing instead of clustering analysis for microstates. Other studies, which for the most part use clustering analysis, report a longer duration (Ihl et al., 1993; Schumacher et al., 2019; Smailovic et al., 2019; Musaeus et al., 2020; Tait et al., 2020). Also, more recent studies using clustering analysis did not find any significant differences (Nishida et al., 2013) or an increased occurrence and coverage for microstate A (Musaeus et al., 2019). In addition, some studies have found an altered features of microstate D in patients with AD (Smailovic et al., 2019; Tait et al., 2020) or that longer duration of microstate D may be a predictor of conversion from MCI to AD (Musaeus et al., 2019). Changes in microstate D has directly been associated with the disruption of the fronto-parietal network (Musaeus et al., 2019).

**FIGURE 2:**
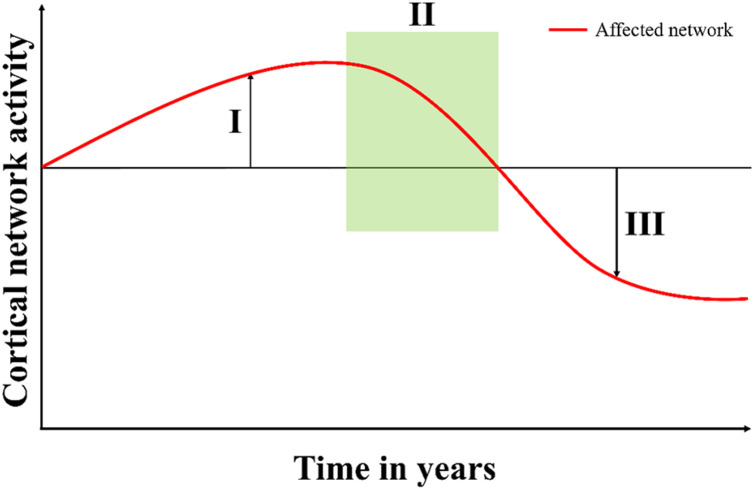
Proposed model of cortical network dysfunction as seen in neurodegenerative diseases. When the neurodegenerative disease starts to affect a cortical network, **(I)** *the compensation phase* shows increased activity in the affected network (microstate D). This continues until the network can no longer compensate and then the patient starts to present symptoms in **(II)** *the symptomatic phase* (time of referral). In **(III)** *the decompensation phase*, the network can no longer compensate for the damages, and the activity falls below the previous level.

A possible explanation is that the diverging findings in patients with AD, may reflect the changes in microstates at different stages of the neurodegenerative disease. In our study, patients with recent onset of symptoms (<1 year) had an increase in microstate D features as compared to a decrease in patients who had symptoms for longer (>2 years), see Figure 1. In support of this, a study found that patients with *CHMP2B*-FTD showed an increase in cerebral blood flow at the initial phase of the disease (Gydesen et al., 1987), which may be a sign of a compensatory upregulation and possibly associated with functional changes as measured with EEG. However, further studies are needed to understand the association between cerebral blood flow and microstate changes. Furthermore, the pre-symptomatic carriers did not show signs of cognitive decline as measured with ACE (see Table 1) but a small increase in duration for microstate D was found in these participants (see Table 2). In addition, studies investigating transgenic mouse models with mutant CHMP2B showed that changes were most pronounced in the frontal areas and suggested a compensatory mechanism of local synchrony to compensate for the breakdown of the cortical networks (Tait, 2019). We therefore propose a biphasic model of cortical network dysfunction in neurodegenerative diseases as seen in microstates (see Figure 2). Here, in the pre-symptomatic phase the affected cortical network (i.e., microstate D) displays a *compensation phase* with increased activity in the affected network (Figure 2I). This continues until the network can no longer compensate, and consequently the patient becomes symptomatic (Figure 2II). This is followed by *the decompensation phase* (Figure 2III) where the network can no longer compensate for the neurodegeneration. In the case of *CHMP2B*-FTD, the frontal and parietal brain areas are affected, and the features of microstate D starts to decline. These findings are of course preliminary and larger prospective studies are needed to support this model including other types of neurodegenerative diseases. However, the *CHMP2B* family represents a homogenous, unique and powerful cohort to generate hypotheses which may be verified in other cohorts including both familial and sporadic cases with neurodegenerative diseases.

For global relative power, we found a trend in the alpha band (*p-*value = 0.099, *F-*value = 2.881). In general, alpha power has been associated with attention (Klimesch, 2012; Benedek et al., 2014), and patients with *CHMP2B*-FTD show problems with attentional control early in the disease. However, no significant differences were found, which is in line with previous studies finding diverging results (Stigsby et al., 1981; Besthorn et al., 1996; Yener et al., 1996; Passant et al., 2005) and the relative alpha was not lower in patients with *CHMP2B*-FTD. Future studies should assess the spectral power changes associated with FTD over time.

We chose to extract four microstates since it is the most commonly reported number of microstates and have been shown to be reliable (Khanna et al., 2014) in spite of the low sample size. We found that the algorithm was robust, and the main findings could be replicated (see Supplementary Material). Furthermore, even though the GEV was not significantly different between the three groups, it was low compared to other studies [normally reporting a GEV of >70% (Michel and Koenig, 2018)]. This could be due to previous studies recording EEGs on younger participants, or that we back fitted the microstates based on concatenated GFP peaks for all participants and did not calculate individual maps.

We acknowledge the limitations of the small sample size, but since the disease is a very rare genetic condition only described in this unique Danish family, it was not possible to recruit more subjects. Overall, the statistical analyses should be carefully interpreted due to the small sample size, which limits the statistical power to observe changes with confidence, However, we found a large effect size (*d* = 0.940), which may point to a difference between the groups. Furthermore, we did not have recent MRI or FDG-PET scans for all participants, which limits our ability to investigate the connection between structural, metabolic and microstate features. In addition, the lack of neuropsychological testing limits us from understanding the relationship between the clinical presentation and microstate features.

## Conclusion

In the present exploratory study, we found evidence of changes in microstate D in patient with *CHMP2B*-FTD. Microstate D has previously been shown to be associated with the fronto-parietal network, which is strongly associated with executive function. Furthermore, we found a pattern of increased activity in microstate D when the symptoms had been present for <1 year, whereas the patients who were symptomatic for >2 years showed decreased network activity. We propose a biphasic model for interpreting microstate changes in *CHMP2B*-FTD, which may be applicable to other neurodegenerative diseases and may explain the conflicting results of network activity found in other neurodegenerative diseases.

## Data Availability Statement

The datasets presented in this article are not readily available because of Danish regulations. Requests to access the datasets should be directed to christian.sandoee.musaeus@regionh.dk.

## Ethics Statement

The studies involving human participants were reviewed and approved by The Ethics Committee of the Capital Region of Denmark (H-1-2012-041). The patients/participants provided their written informed consent to participate in this study.

## The FReJA Consortium

Gydesen S^d^, Brown J^e^, Isaacs AM^f^, Collinge J^g^, Gade A^h^, Englund E^i^, Fisher E^f^, Nielsen TT^a^, Thusgaard T^j^, and Holm I^k^.

^a^ Danish Dementia Research Centre (DDRC), Rigshospitalet and Department of Clinical Medicine, University of Copenhagen, Copenhagen, Denmark
^d^ Psychiatric Centre Ballerup, Copenhagen University Hospital, Ballerup, Denmark
^e^ Department of Neurology, Addenbrooke’s Hospital, Cambridge, United Kingdom
^f^ Department of Neurodegenerative Disease and UK Dementia Research Institute at UCL, UCL Queen Square Institute of Neurology, London, United Kingdom
^g^ Institute of Prion Diseases and MRC Prion Unit at UCL, Courtauld Building, London, United Kingdom
^h^ Department of Psychology, University of Copenhagen, Copenhagen, Denmark
^i^ Department of Pathology, University Hospital, Lund, Sweden
^j^ Parkvaenget Nursing Home, Holstebro, Denmark
^k^ Department of Pathology, Aalborg University Hospital, Aalborg, Denmark

## Author Contributions

CM, JP, PR, and JN initiated the study, recruited patients, and gathered patient data. TK, MH, and TB were responsible for equipment including recording setup. CM and PR analyzed the data. CM wrote the first draft of the article. All authors have edited and critically revised the manuscript.

## Conflict of Interest

The authors declare that the research was conducted in the absence of any commercial or financial relationships that could be construed as a potential conflict of interest.

## Publisher’s Note

All claims expressed in this article are solely those of the authors and do not necessarily represent those of their affiliated organizations, or those of the publisher, the editors and the reviewers. Any product that may be evaluated in this article, or claim that may be made by its manufacturer, is not guaranteed or endorsed by the publisher.
