# Supplementary material for: Cortical Frontoparietal Network Dysfunction in CHMP2B-Frontotemporal Dementia
Source: Front Aging Neurosci. 2021 Sep 13;13:714220. doi: 10.3389/fnagi.2021.714220 (PMC8475188; doi:10.3389/fnagi.2021.714220)
Supplement: Supplementary file 1 [file Data_Sheet_1.docx]

**Supplementary material**

See page 2-4 for repeated analysis of microstate analysis.

See page 5 for global maps for the main analysis.

See page 6 for the global relative power results.

See page 7 for the results from the microstate features for microstates A-D for the main analysis.

**Repeating the analysis to test whether it is robust**

To test whether the calculations were robust, we repeated the entire analysis three times, see below.
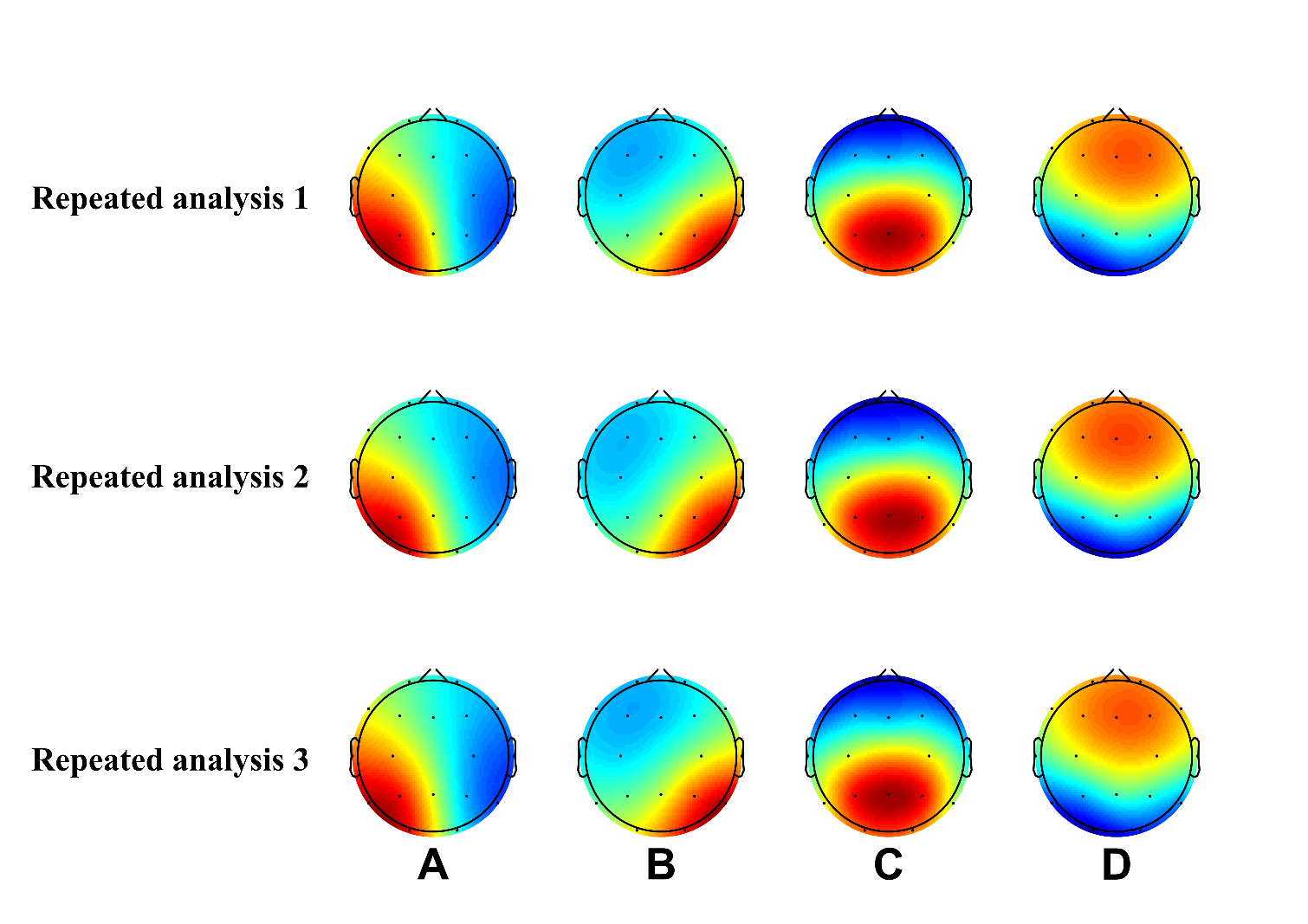


**Supplemental figure 1**: Overview of the maps from the three repeated analyses.

*Repeated analysis 1*

**Supplemental table 1**
Duration, occurrence, and coverage for each microstate (A-D), p-value and F-value for the comparison between HC, pre-symptomatic mutation carriers, and symptomatic FTD-3 patients for the repeated analysis 1.

Abbreviations: HC = healthy controls; FTD3 = Frontotemporal dementia type 3. P-values show the differences when comparing patients with FTD-3, pre-symptomatic carriers, and HC.

*Repeated analysis 2*

**Supplemental table 2**
Duration, occurrence, and coverage for each microstate (A-D), p-value and F-value for the comparison between HC, pre-symptomatic mutation carriers, and symptomatic FTD-3 patients for the repeated analysis 2.

Abbreviations: HC = healthy controls; FTD3 = Frontotemporal dementia type 3. P-values show the differences when comparing patients with FTD-3, pre-symptomatic carriers, and HC.

*Repeated analysis 3*

**Supplemental table 3**
Duration, occurrence, and coverage for each microstate (A-D), p-value and F-value for the comparison between HC, pre-symptomatic mutation carriers, and symptomatic FTD-3 patients for the repeated analysis 3.

Abbreviations: HC = healthy controls; FTD3 = Frontotemporal dementia type 3. P-values show the differences when comparing patients with FTD-3, pre-symptomatic carriers, and HC.

**Supplementary figure 2**: Global maps (A-D) calculated based on the aggregated dataset from all participants and back-fitted to each EEG recording.


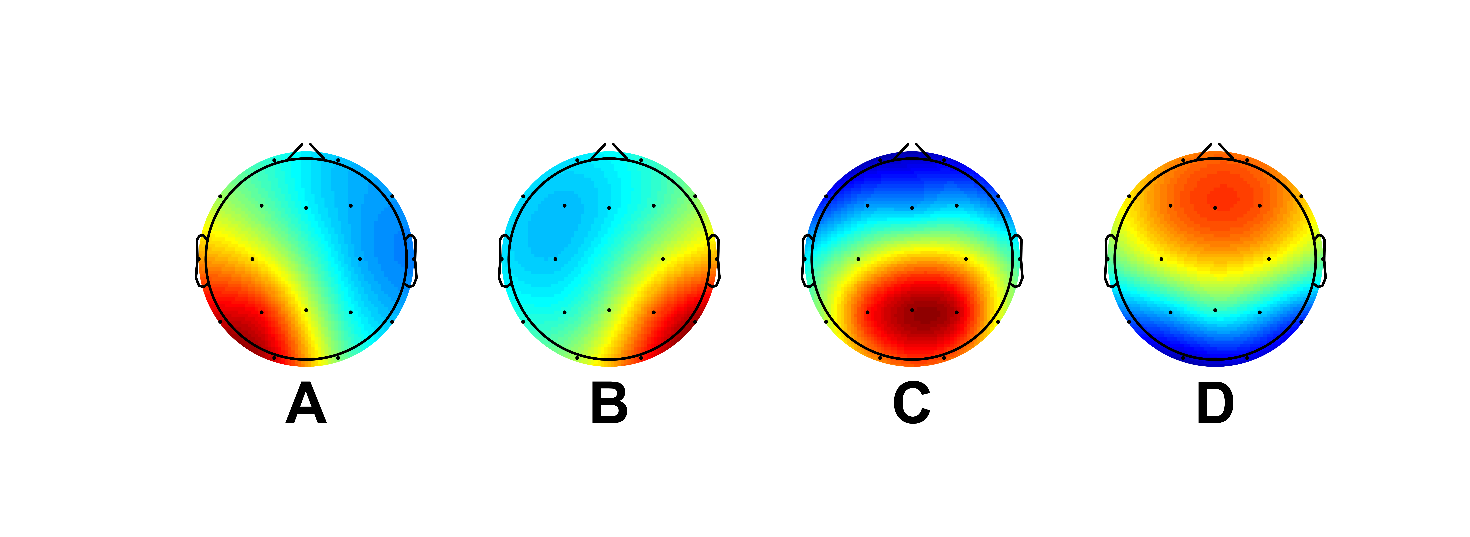


**Supplementary Figure 3**: Global relative power for each frequency band for symptomatic CHMP2B-FTD patients, pre-symptomatic mutation carriers and non-carrier controls. Error bars represent the standard deviation.

**
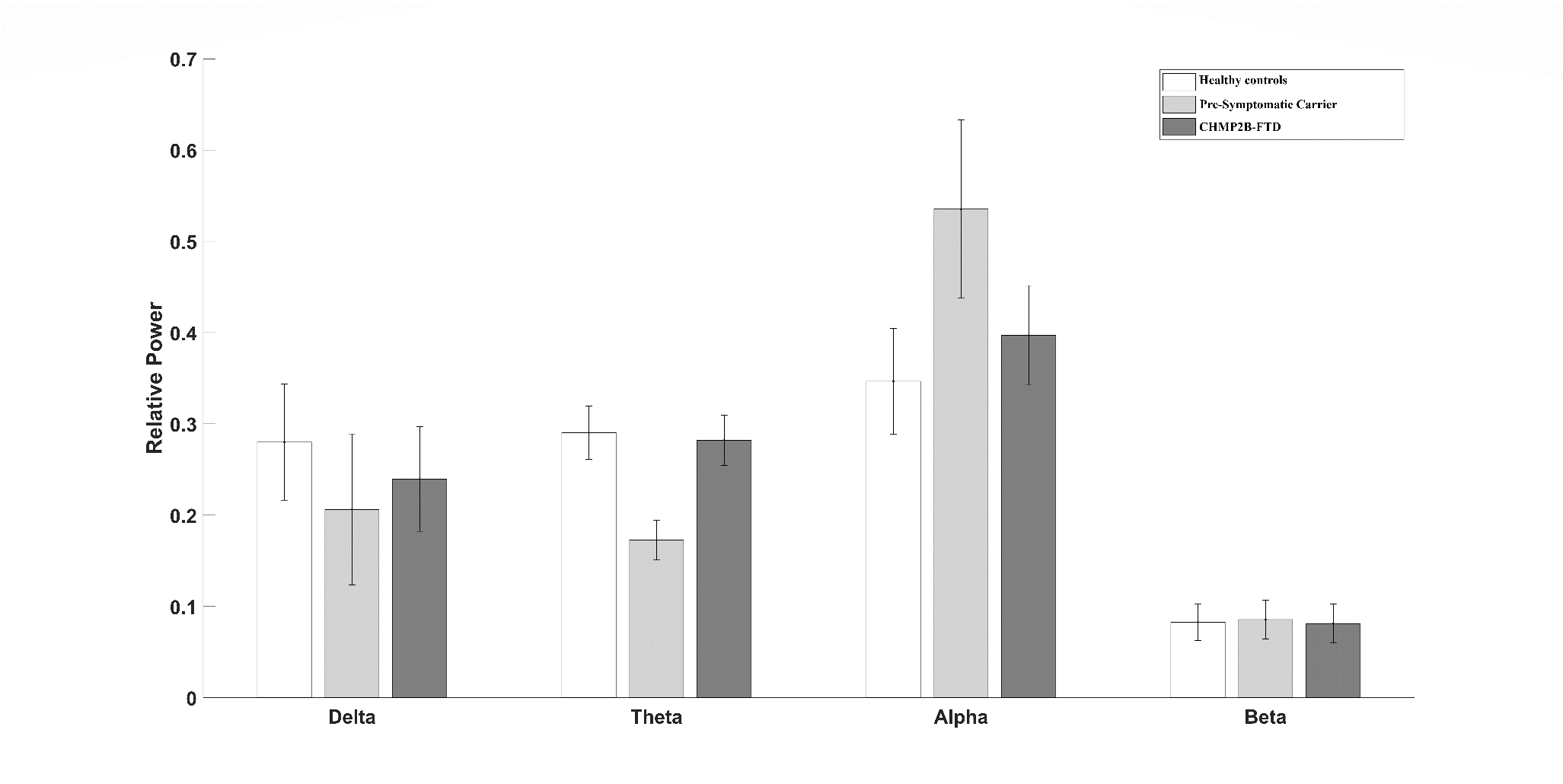
**

**Supplementary table 4.** Microstate features for microstates A-D for controls, CHMP2B-FTD patients with recent symptom onset (<1 year) and patients who had been symptomatic for longer periods (>2 years)

**Controls**

**FTD >2 years**

**FTD <1 year**

**Controls**

**FTD >2 years**

**FTD <1 year**

**Controls**

**FTD >2 years**

**FTD <1 year**

**Microstate A**

77.75 (4.56)

93.43 (14.88)

84.48 (31.19)

2.80 (0.46)

3.35 (0.66)

1.93 (0.09)

21.76 (3.54)

31.93 (11.36)

16.49 (6.81)

**Microstate B**

77.48 (4.97)

79.25 (3.73)

80.30 (16.43)

2.73 (0.40)

2.99 (0.22)

2.31 (0.65)

21.25 (4.02)

23.65 (1.93)

18.04 (1.45)

**Microstate C**

82.91 (8.27)

85.21 (12.89)

84.42 (6.06)

3.14 (0.51)

2.89 (0.56)

2.64 (0.86)

26.13 (5.85)

25.04 (7.77)

22.06 (5.67)

**Microstate D**

91.92 (10.86)

75.91 (8.90)

133.37 (41.24)

3.36 (0.16)

2.53 (0.28)

3.41 (1.03)

30.86 (3.67)

19.38 (4.51)

43.41 (0.31)

**Duration**

**Occurrence**

**Coverage**

Abbreviations: FTD >2 years = patients with CHMP2B-FTD who have been symptomatic for more than 2 years; FTD >2 years = patients with CHMP2B-FTD who have been symptomatic for more less than 1 year.
